# The influence of schooling on performance in the Mattis Dementia Rating Scale (DRS)

**DOI:** 10.1590/S1980-57642010DN40200009

**Published:** 2010

**Authors:** Cláudia Sellitto Porto, Paulo Caramelli, Ricardo Nitrini

**Affiliations:** 1 PhD, Behavioral and Cognitive Neurology Unit, Department of Neuroly of University São Paulo School of Medicine and Cognitive Disorders Reference Center (CEREDIC), Hospital das Clínicas of University São Paulo School of Medicine, São Paulo SP, Brazil.; 2 MD, PhD, Behavioral and Cognitive Neurology Unit, Department of Internal Medicine, Faculty of Medicine, Federal University of Minas Gerais, Belo Horizonte MG, Brazil.; 3 MD, PhD, Behavioral and Cognitive Neurology Unit, Department of Neuroly of University São Paulo School of Medicine and Cognitive Disorders Reference Center (CEREDIC), Hospital das Clínicas of University São Paulo School of Medicine, São Paulo SP, Brazil.

**Keywords:** Alzheimer’s disease, neuropsychological assessment, schooling

## Abstract

Studies have shown the influence of schooling on performance in the Dementia Rating Scale (DRS), suggesting that a single cut-off score is not appropriate for all groups of elderly people.

**Objectives:**

To verify the influence of schooling on the DRS in a Brazilian elderly population.

**Methods:**

The DRS was applied to 118 cognitively healthy controls and to 97 patients with mild probable Alzheimer’s disease (AD). In order to analyze the influence of education, patients and controls were divided into four groups of schooling (GRSC): GRSC 1 with 1 to 4 years of schooling, GRSC 2 with 5 to 8 years of schooling, GRSC 3 with 9 to 11 years of schooling, and GRSC 4 with more than 11 years of schooling.

**Results:**

In the intragroup analysis, the performance of controls within each schooling group was compared, revealing a significant difference on total score and the subscales Attention, I/P and Conceptualization. The same procedure was used for the AD patients and a significant difference was observed for total score and the subscales Attention, Construction, Conceptualization and Memory. In the intergroup analysis, the results on total DRS and for the I/P, Conceptualization and Memory subscales showed significant differences in GRSC 1, 2, 3 and 4. The Attention subscale showed differences in GRSC 3 and 4, and on the Construction subscale in GRSC 1 and 4.

**Conclusions:**

The results highlight the importance of norms for the DRS in the Brazilian population that take into account the effects of schooling on the scores of this scale.

## Abstract (pt)

Estudos recentes têm demonstrado a influência da escolaridade no desempenho da Escala de Avaliação de Demência (DRS) sugerindo que uma única nota de corte não é apropriada para todos os grupos de pessoas idosas.

**Objetivos:**

Verificar a influência da escolaridade na DRS para uma população brasileira de idosos.

**Métodos:**

A DRS foi aplicada em 118 controles cognitivamente saudáveis e em 97 pacientes com doença de Alzheimer (DA) leve. Para a análise da influência da escolaridade, pacientes e controles foram separados em quarto grupos de escolaridade (GRESC): GRESC 1 com 1 a 4 anos de escolaridade, GRESC 2 com 5 a 8 anos de escolaridade, GRESC 3 com 9 a 11 anos de escolaridade, e GRESC 4 com mais de 11 anos de escolaridade.

**Resultados:**

Na análise intragrupo o desempenho de controles, em cada grupo de escolaridade, foi comparado e diferenças significativas foram estabelecidas no escore total e nas subescalas Atenção, I/P e Conceituação. O mesmo procedimento foi realizado com o grupo de pacientes com DA e diferenças significativas foram observadas no escore total e nas subscalas Atenção, Construção, Conceituação e Memória. Na análise intergrupos o resultado no escore total e nas subescalas I/P, Conceituação e Memória mostraram diferenças significativas no GRESC 1, 2, 3 e 4. A subescala Atenção mostrou diferenças no GRESC 3 e 4 e a subescala Construção nos GRESC 1 e 4.

**Conclusões:**

Os resultados mostraram a importância de normas de escolaridade apropriadas para a DRS na população brasileira levando-se em consideração os efeitos da escolaridade nos escores desta escala.

The Mattis Dementia Rating Scale (DRS)^1,2^ is used for the assessment of general cognitive status and has frequently been adopted in both clinical practice and research. It is easy to apply and quick to administer, taking about 30 to 40 minutes in patients with dementia. Its 36 tasks are grouped into five subscales, each evaluating a different cognitive area, namely attention, initiation/perseveration (I/P), construction, conceptualization and memory.

In comparison with other brief batteries, the DRS presents some advantages: it provides more detailed information about the cognitive functions that are impaired or preserved, since it performs a more in-depth evaluation of a greater number of cognitive domains,^3,4^ and also has greater sensitivity in the diagnosis of Alzheimer’s disease (AD).^5-8^

Schooling, age and cultural factors interfere in test accuracy, pointing to the importance of adequate norms for different populations. Recent studies have shown the influence of age and schooling on performance in the DRS, suggesting that a single cut-off score is not appropriate for all groups of elderly people.^6,9-11^

In the Brazilian population, Porto et al.^12^ have demonstrated the value of the DRS in the differential diagnosis between mild AD and cognitively healthy controls and highlighted the importance of norms for this scale in the Brazilian population which take into account the effects of age and education. In this sample population, the effects of education were more evident than the effects of age.

Another study involving the Brazilian population analyzed the influence of low schooling and illiteracy on DRS performance in a normal elderly group, showing that illiterate individuals had lower DRS total scores and subscale scores than did literate subjects.^13^

Studies have emphasized the effect of schooling on the DRS total score but few have described how different grades of schooling influence the DRS subscales. The main objective of this study was therefore to verify the influence of schooling on the DRS for Brazilian healthy elderly and to compare the results with a mild AD group.

## Methods

This study involved 118 control subjects, aged 51 to 84 years (mean=69.37±8.00), with 1 to 16 years of schooling (mean=9.25±4.91), comprising 79 women and 39 men; and a group of 97 patients with probable AD and mild dementia, aged 53 to 88 years (mean 72.39±7.85), comprising 61 women and 36 men, with schooling ranging from 1 to 16 years (mean=9.39±4.94).

The diagnosis of mild dementia was based on the criteria of the Diagnostic and Statistical Manual of Mental Disorders, Third Edition, revised (DSM-III-R) ^14^ and the diagnosis of probable AD was based on the criteria developed by the National Institute of Neurological Diseases and Communicative Disorders and Stroke-Alzheimer’s Disease and Related Disorders Association (NINCDS-ADRDA).^15^ All patients were attended by members of the Behavioral and Cognitive Neurology Unit of the Department of Neurology of the University of São Paulo School of Medicine, Brazil, and were submitted to extensive neuropsychological assessment, neurological examination, laboratory testing and neuroimaging (computed tomography (CT) or magnetic resonance (MR) of the skull).

The neuropsychological evaluation consisted of the Mini-Mental State Examination (MMSE),^16,17^ and tests to evaluate visual and verbal memory (Visual Reproduction - Wechsler Memory Scale (WMS),^18^ Rey Complex Figure memory,^9^ Logical Memory - WMS,^18^ Rey Auditory Verbal Learning Test,^20^ constructive skills (Block Design - Wechsler Intelligence Adult Scale-WAIS,^21^ Rey Complex Figure copy,^19^ visual perception (Raven Colored Matrices^22^ or Hooper Visual Organization Test ^23^) , language (Boston Naming Test )^24^ and executive functions (Trail Making Test^25^ and phonemic verbal fluency (F.A.S.). ^25^

The control group was composed of spouses or consorts of the patients, and volunteers from the community, who presented no memory disorders and were fully independent in terms of daily activities. The information for inclusion or exclusion of the controls was obtained via a semi-structured interview, conducted by the researcher (CSP) prior to the application of the DRS. The researcher questioned the interviewees about their memory, daily activities, medications, and history of depression, brain injury, stroke, *diabetes mellitus* and high blood pressure. The Mini-Mental State Examination (MMSE) was applied to all individuals. Subjects with neurological diseases, history of alcoholism, depression, other psychiatric disorders, non-corrected visual or auditory disorders, motor disorders, or users of psychotropic drugs that could affect cognitive functions were excluded. Chronic diseases such as arterial hypertension, *diabetes mellitus* and heart diseases, when under good control, did not prevent participation in the study.

In order to investigate the influence of schooling on DRS performance, patients and controls were divided into four schooling groups: group of schooling (GRSC) 1 with 1 to 4 years of schooling, GRSC 2 with 5 to 8 years of schooling, GRSC 3 with 9 to 11 years of schooling, and GRSC 4 with more than 11 years of schooling. GRSC 1 consisted of 30 patients with AD (mean age=73.03±7.21; mean schooling =3.50±0.94, 23 women and 7 men) and 38 control subjects (mean age=70.68±7.07; mean schooling=3.50±0.16; 28 women and 10 men); GRSC 2, 17 patients with AD (mean age=73.29±8.61; mean schooling=7.59±0.71; 11 women and 6 men) and 21 controls (mean age=70.57± 7.92; mean schooling=7.38±1.07; 14 women and 7 men); GRSC 3, 22 AD ( mean age=72.59±7.56; mean schooling= 10.64±0.66; 15 women and 7 men) and 22 controls (mean age=67.86±7.79; mean=10.91±0.29; 18 women and 4 men); and, GRSC 4, 28 patients with AD (mean age= 71.00±8.46; mean schooling=15.82±0.65; 12 women and 16 men) and 37 controls (mean age=68.24±9.00; mean schooling=15.24±1.01; 19 women and 18 men).

The DRS was applied to all subjects in a single individual session and in the order recommended by the author. The DRS tasks are presented in a fixed order, and only the Attention tests are not grouped in a sequence, as they also serve as distractors to the Memory subscale. Within each subscale, the most difficult tests are presented in first and second place, and if performed well, subsequent items in the subscale are scored with correct performance. The advantage of this procedure is that it permits the shortening of the total test time for individuals whose cognitive function is better preserved.

The number of points credited for the correct response varies in accordance with the tasks, while the total points in each subscale score provides a partial score for that subscale. The partial scores are: attention, 37 points; initiation/perseveration (I/P), 37 points; construction, 6 points; conceptualization, 39 points; and memory, 25 points. The maximum total possible score is 144 points.

All participants signed written informant consent terms and the study was approved by the Ethics Committee of the Hospital das Clínicas of the University of São Paulo School of Medicine.

### Statistical analyses

Descriptive statistical analyses (mean and standard deviation) were performed for demographic data. Analysis of associations among categorical variables was performed using the chi-square test. When the variables were continuous the comparison were made using the Mann-Whitney test for two samples, and the Kruskall-Wallis test for more than two samples. The level of significance adopted for all analyses was 0.05. All statistical analyses were carried out using the program Statistical Package for the Social Sciences (SPSS), 10.0.

## Results

No significant differences were found between performance by AD and controls on the DRS in relation to gender (p=0.53) and schooling (p=0.79), but a significant difference was found for age (p=0.003).

The mean total score on the DRS for the AD patients was 112.07±12.36, and for the control group, 134.10±8.34. A significant difference was found in relation to the mean total score between the AD group and the controls (p<0.001) and on all the subscales.

No significant differences between AD patients and controls on DRS performance was detected in relation to gender on analyses of the four levels of schooling. However, a significant difference was found between AD patients and controls in relation to age in GRSC 3 but not in GRSC 1, 2 and 4. Regarding the schooling variable for AD and controls, no significant difference emerged for GRSC 1, 2 and 3. However, a significant difference between AD patients and controls was found in GRSC 4.

The performance of the control group within each group by years of schooling was compared and a significant difference observed for the subscales Attention (p=0.001), I/P (p<0.001), Conceptualization (p=0.017) and total score (p<0.001). The same procedure was performed for the AD patients group and a significant difference was found for the subscales Attention (p=0.018), Construction (p=0.016), Conceptualization (p<0.001), Memory (p=0.046) and for total score (p<0.001), but not on the subscale I/P (p=0.060).(Table 1)

**Table 1:** Intragroups analysis.

	Attention	I/P	Construction	Conceptualization	Memory	Total
AD	0.001	<0.001	0.582	0.017	0.086	<0.001
Controls	0.018	0.060	0.016	<0.001	0.046	<0.001

AD: Alzheimer's disease; I/P: initiation/perseveration; p<0.05

Table 2 shows the performance of AD patients and controls on the DRS (total score and subscales) for the four levels of schooling.

**Table 2:** Performance of AD patients and controls by schooling group on DRS total and subscales.

DRSN	GRSC 1 (1-4)			GRSC 2 (5-8)			GRSC 3 (9-11)			GRSC 4 (>11)	
	AD	Controls		AD	Controls		AD	Controls		AD	Controls
	30	38		17	21		22	23		28	37
Attention											
Mean	33.96	34.65		35.35	35.57		34.00	36.13		35.10	36.00
SD	2.00	1.93		1.61	1.56		2.04	1.42		1.44	1.02
p	0.138			0.622			<0.001			0.011	
I/P											
Mean	26.93	33.57		31.35	34.52		29.31	34.54		28.75	36.45
SD	5.45	4.08		4.44	3.28		5.90	4.05		5.63	1.36
p	< 0.001			0.016			0.001			<0.001	
Construction											
Mean	5.03	5.76		5.94	5.95		5.77	5.90		5.46	5.86
SD	1.27	0.67		0.24	0.21		0.52	0.29		1.07	0.67
p	0.004			0.954			0.365			0.026	
Conceptualization											
Mean	26.73	33.07		28.35	34.28		28.31	35.45		33.78	35.97
SD	5.43	5.25		5.39	4.11		6.46	4.02		4.61	3.85
p	< 0.001			0.001			<0.001			0.028	
Memory											
Mean	12.30	22.84		14.47	22.71		14.09	23.59		15.03	23.64
SD	3.38	1.98		3.20	2.62		3.91	2.80		4.30	1.43
p	< 0.001			<0.001			<0.001			<0.001	
Total											
Mean	104.96	130.05		115.35	133.04		111.50	135.63		118.14	137.94
SD	11.17	8.93		10.11	9.32		12.51	7.65		11.20	5.20
p	<0.001			<0.001			<0.001			<0.001	

I/P, initiation/perseveration; SD, standard deviation. Mann-Whitney Test (p<0.05).

## Discussion

In the intragroup analysis, significant differences were observed among controls across the four different schooling groups for the subscales attention, I/P, conceptualization and total score.

The DRS total score in GRSC 1 was 130.05±8.93 and in the GRSC 4 137.94±5.20, showing the influence of schooling on this scale.

The attention subscale is composed by Digit Span (Forward and Backward), concentration/attention, answers to two commands, word list reading, and similarity of figures, tests considered easy to perform. Bennett et al. affirmed in their study that performance on the DRS attention scale was preserved across education levels in their population of octogenarians and nonagenarians.

Both I/P and conceptualization subscales were significantly influenced by schooling in all subgroups. The influence of schooling on verbal fluency, presented in the I/P subscale and accounting for more than half of the subscale total, was observed in studies carried out by Brucki^26^ and Caramelli et al.^27^ The conceptualization subscale contains tasks related to semantic memory. Porto et al.^12^ detected no influence of schooling among healthy individuals with different levels of education on the scores of this subscale.

In the analysis of patients with AD for the four groups of schooling, a significant difference was found in the subscales attention, construction, conceptualization, memory and for overall DRS score.

In our sample, GRSC 3 and 4 demonstrated difficulties in the attention subscale. GRSC 3 showed differences between AD patients and controls for age, and GRSC 4 for schooling . These results deserve further investigation as they diverge from findings generally reported in the literature.^9^

The findings of Hohl et al.^28^ indicated that Hispanic AD patients performed significantly worse than non-Hispanics in terms of total DRS score, and scores on the DRS subscales for conceptualization and memory.

The construction subscale, considered to have low sensitivity to the effects of age and schooling,^9^ appeared to be influenced by low levels of schooling. The tasks in this subscale entail copying geometrical figures and name writing, which although relatively easy become more complex for individuals with very low levels of schooling. Individuals with higher educational level also presented impairment in the construction subscale and we are unable to satisfactorily explain these results in view of the ease of these tests.

The small sample size as well as the differences in demographics among the schooling groups, represent limitations of the current study. However, the results show the importance of normative values for the DRS in the Brazilian elderly population that take into account the effects of schooling on test performance. Moreover, our results reaffirm that the diagnosis of dementia based on neuropsychological assessment must be made with caution in individuals with low educational level.

## References

[1] Mattis S, Bellak L, Karasu TB (1976). Mental status examination for organic mental syndrome in the
elderly patient. Geriatric psychiatry: a handbook for psychiatrists and primary care
physicians.

[2] Mattis S (1988). Dementia Rating Scale: professional manual.

[3] Salmon DP, Thal LJ, Butters N, Heindel WC (1990). Longitudinal evaluation of dementia of the Alzheimer type: a
comparison of 3 standardized mental status examinations. Neurology.

[4] Karbe H, Kertesz A, Davis J, Kemp BJ, Prato FS, Nicholson RL (1994). Quantification of functional deficit in Alzheimer's disease using
a computer-assisted mapping program for Tc-HMPAO SPECT. Neuroradiology.

[5] Paolo AM, Trostr AI, Glatt SL, Hubble JP, Koller WCJ (1995). Differentiation of the dementias of Alzheimer's and Parkinson's
disease with the dementia rating scale. Geriatr Psychiatry Neurol.

[6] Lukatela K, Cohen R, Kessler H (2000). Dementia Rating Scale performance: a comparison of vascular and
Alzheimer's dementia. J Clin Ex Neuropsychol.

[7] Paulsen JS, Butters N, Sadek JR (1995). Distinct cognitive profiles of cortical and subcortical dementia
in advanced illness. Neurology.

[8] Kertesz A, Clydesdale S (1994). Neuropsychological deficits in vascular dementia vs Alzheimer's
disease. Arch Neurol.

[9] Bennett A, Nadler J, Spigler M (1997). The Mattis Dementia Rating Scale in nursing home octagenarians
and nonagenarians: effects of age and education. J Geriatr Psychiatry Neurol.

[10] Bank AL, Yochim BP, MacNeill SE, Lichtenberg PA (2000). Expanded Normative Data for the Mattis Dementia Rating Scale for
Use with Urban, Elderly Medical Patients. Clin Neuropsychol.

[11] Schmidt R, Freidl W, Fazekas F (1994). The Mattis Dementia Rating Scale: normative data from 1,001
healthy volunteers. Neurology.

[12] Porto CS, Charchat-Fishman H, Caramelli P, Bahia VS, Nitrini R (2003). Brazilian version of the Mattis Dementia Rating Scale: diagnosis
of mild dementia in Alzheimer's disease. Arq Neuropsiquiatr.

[13] Foss MP, Vale Fde A, Speciali JG (2005). Influence of education on the neuropsychological assessment of
the elderly: application and analysis of the results from the Mattis
Dementia Rating Scale (MDRS). Arq Neuropsiquiatr.

[14] American Psychiatric Association (1987). Diagnostic and statistical manual of mental disorders.

[15] Mckhann G, Drachman D, Folstein M, Katzman R, Price D, Stadlan EM (1984). Clinical diagnosis of Alzheimer's disease: report of the
NINCDS-ADRDA work group under the auspices of department of health and human
services task force on Alzheimer's disease. Neurology.

[16] Folstein MF, Folstein SE, McHugh PR (1975). "Mini-mental state": a practical method for grading the cognitive
state of patients for the clinician. J Psychiatr Res.

[17] Brucki SMD, Nitrini R, Bertolucci PHP, Caramelli P, Okamoto IH (2003). Normas sugeridas para o uso do Mini-Exame do Estado Mental (MEEM)
em nosso meio. Arq Neuropsiquiatr.

[18] Wechsler D (1987). Wechsler Memory Scale: manual.

[19] Rey A (1998). Figuras Complexas de Rey.

[20] Diniz LFM, Cruz MF, Torres VM, Consenza RM (2000). O teste de aprendizagem auditivo-verbal de Rey: normas para uma
população brasileira. Rev Bras Neurol.

[21] Wechsler D (1993). Teste de Inteligência para adultos (WAIS). Manual.

[22] Raven JC, Raven J, Court JH (1988). Manual matrizes progressivas coloridas.

[23] (1983). Hooper Visual Organization Test (VOT) Manual.

[24] Radanovic M, Mansur LL, Scaff M (2004). Normative data for the Brazilian population in the Boston
Diagnostic Aphasia Examination: influence of schooling. Braz J Med Biol Res.

[25] Spreen O, Strauss E (1998). A compendium of neuropsychological tests: administration, norms, and
commentary.

[26] Brucki SMD (1996). Dados Normativos para o uso do teste de fluência verbal
(categorias animais) em nosso meio.

[27] Caramelli P, Carthery MT, Porto CS, Charchat-Fichman H, Bahia V, Nitrini R (2001). Análise qualitativa da fluência verbal no
envelhecimento normal e na doença de Alzheimer: efeitos da
escolaridade. Arq Neuropsiquiatr.

[28] Hohl U, Grundman M, Salmon DP, Thomas RG, Thal LJ (1999). Mini-Mental State examination and Mattis Dementia Rating Scale
performance differs in Hispanic and non-Hispanic Alzheimer's disease
patients. J Int Neuropsychol Soc.

